# Zero-Field and Field-Induced Interactions between Multicore Magnetic Nanoparticles

**DOI:** 10.3390/nano9050718

**Published:** 2019-05-09

**Authors:** Andrey A. Kuznetsov

**Affiliations:** 1 Institute of Continuous Media Mechanics UB RAS, Perm Federal Research Center UB RAS, Perm 614013, Russia; kuznetsov.a@icmm.ru; 2 Physics of Phase Transitions Department, Perm State University, Perm 614990, Russia

**Keywords:** multicore magnetic nanoparticles, magentic nanoclusters, magnetic interactions, Langevin dynamics simulations

## Abstract

In this paper, the Langevin dynamics simulation method is used to study magnetic interactions between a pair of multicore magnetic nanoparticles subjected to a uniform magnetic field. Multicore nanoparticles are modelled as spherical rigid clusters of single-domain superparamagnetic cores coupled via dipole-dipole interactions. It is shown that the magnetic force between two well-separated clusters in a strong applied field can be accurately described within the induced point-dipole approximation. However, this approximation also assumes that there are no interactions between clusters in the zero-field limit. On the contrary, simulations indicate the existence of a relatively small attractive magnetic force between clusters, even in the absence of an applied field. It is shown that this force is a direct superparamagnetic analog of the van der Waals interaction between a pair of dielectric spheres.

## 1. Introduction

Multicore magnetic nanoparticles (MCMNPs) are clusters of single-domain magnetic nanocrystals (“*cores*”) embedded in a rigid non-magnetic matrix [1,2]. Iron oxide cores with characteristic linear sizes of the order of 10 nm are probably the most common case. The size of MCMNPs themselves can range from tens to a few hundred nanometers. Recently, MCMNPs have attracted a lot of research attention due to their exceptionally wide range of potential biomedical and biotechnological applications [3]. They are considered to be prominent in such areas as magnetic bioseparation and immunoassays [4,5,6], targeted drug delivery [7,8], magnetic resonance imaging [9,10,11], magnetic particle imaging [12,13,14], and magnetic hyperthermia [15,16,17,18].

Theoretical and numerical investigations of the last decade have firmly established that the response of an isolated MCMNP to an applied magnetic field can be significantly affected by magnetic interactions (dipole-dipole and/or exchange ones) between cores within the MCMNP. Such *intracluster* interactions influence the equilibrium magnetic moment [19,20,21], magnetization dynamics [22,23,24], and magnetophoretic mobility [25] of MCMNPs. However, so far, relatively little attention has been paid to the problem of interactions between separate MCMNPs—that is, *intercluster* interactions. At the same time, collective phenomena in ensembles of other types of magnetic particles is an active and ever-expanding area of research. One notable example is single-core magnetic nanoparticles (SCMPNs). They consist of a single-domain magnetic core surrounded by a non-magnetic protective shell. The core has a magnetic moment of constant magnitude, but typically, due to thermal fluctuations, its averaged projection in any direction is zero in the absence of an applied field. This phenomenon is known as superparamagnetism [26]. Qualitatively, the superparamagnetic medium behaves similarly to the atomic paramagnet, although the magnetic moments which one is dealing with are the moments of ferromagnetic domains and not that of single atoms. Magnetic interactions greatly increase the static magnetic susceptibility of an SCMNP ensemble [27] and change the spectrum of its dynamic susceptibility [28]. If SCMNPs are dispersed in a liquid or soft elastic matrix, long-range and anisotropic dipole-dipole interactions lead to the formation of various aggregates, even in a zero field. Depending on the concentration and interaction energy, SCMNPs can self-assemble into chains, rings, branched structures, and three-dimensional percolating networks [29,30,31,32,33]. Other examples of well-studied systems in which magentic interparticle interactions play a pivotal role are magnetorheological fluids and elastomers, that is, ensembles of magnetizable microparticles dispersed in a liquid or polymer matrix, respectively [34]. Such microparticles are also commonly referred to as “microspheres” or “beads”. In zero fields, beads have negligible magnetization and do not interact, but the applied field gives rise to a magnetic interbead interaction. It can be approximated by an interaction between induced point-dipoles if the interbead separation is large enough. In close contact, however, beads are non-uniformly magnetized, and their interaction becomes much more complex [35,36]. Regardless of whether the Brownian motion of beads is taken into account, the magnetic field always forces them to assemble into chains. Chains, in turn, tend to form thick columnar structures via the so-called lateral chain aggregation [37,38,39].

To date, scarce theoretical studies of collective phenomena in MCMNP ensembles have modelled these nano-objects simply as interacting paramagnetic spheres with zero residual magnetization (i.e., in the same fashion as one models microsized magnetic beads dispersed in a magnetorheological fluid) [40,41,42,43]. The aim of this study was to test the validity of such an approach using the Langevin dynamics simulation method. We will investigate in detail the simplest possible system in which intercluster interactions are present—that is, an isolated pair of MCMNPs in a uniform field.

## 2. Model and Methods

Here, each MCMNP is modeled as a spherical cluster of *N* identical magnetic cores. Most simulations were performed for N=100. Further on, the term “*cluster*” will be used to denote our model of an MCMNP. Each magnetic core in the cluster is modeled as a magnetic sphere of diameter *d* and magnetic moment $\vec{m}$ (the core magnetic moment has a constant magnitude *m*). Cores are superparamagnetic—that is, their magnetic moments $\vec{m}$ can freely rotate within the core body under the influence of magnetic fields and thermal fluctuations. The volume fraction of cores within the cluster is

(1) $$\varphi =\frac{vN}{V}=\frac{d^{3}N}{D^{3}},$$

where $v=\pi d^{3}/6$ is the core volume, $V=\pi D^{3}/6$ is the cluster volume, and *D* is the cluster diameter. For given values of *N* and φ, the cluster is generated as follows. First, the value of *D* is calculated from Equation (1); then, all *N* cores are randomly placed within a spherical volume *V*, one by one, without overlapping. After this procedure is done, positions of cores within the cluster are fixed, in that no translational movement within the cluster volume is allowed (but magnetic moments of cores can still rotate, as was stated above). It is now becoming widely accepted that properties of very densely packed MCMNPs (also known as “nanoflowers”) are substantially determined by the exchange coupling between neighbouring cores [2,44,45]. Here, however, exchange interactions will not be taken into account, and only moderately concentrated clusters with φ≤30% will be considered. Thus, the only magnetic interaction between cores is the dipole-dipole one. The total magnetostatic energy of the *i*-th core is given by

(2) $$\begin{array}{c} u_{i}=u_{i}^{z}+\sum_{j,j\neq i}^{2N}u_{ij}^{dd}, \end{array}$$

(3) $$\begin{array}{c} u_{i}^{z}=-\mu_{0}\left({\vec{m}}_{i}\cdot \vec{H}\right), \end{array}$$

(4) $$\begin{array}{c} u_{ij}^{dd}=-\frac{\mu_{0}}{4\pi }\left[\frac{3\left({\vec{m}}_{i}\cdot {\vec{r}}_{ij}\right)\left({\vec{m}}_{j}\cdot {\vec{r}}_{ij}\right)}{r_{ij}^{5}}-\frac{{\vec{m}}_{i}\cdot {\vec{m}}_{j}}{r_{ij}^{3}}\right], \end{array}$$

where $u_{i}^{z}$ is the Zeeman energy in an applied magnetic field $\vec{H}$, $\mu_{0}$ is the magnetic constant, $u_{ij}^{dd}$ is the energy of dipole-dipole interaction between cores *i* and *j*, and ${\vec{r}}_{ij}$ is the vector between centers of cores *i* and *j*. The summation is over all cores in the system, regardless of whether they belong to the same cluster or not. The total number of cores is the number of cores per cluster (*N*) times the total number of clusters (two within this work). To characterize the ratios between magnetic energy scales and the thermal energy $k_{B}T$, two standard dimensionless parameters are used, namely, the Langevin parameter (dimensionless applied field)

(5) $$\xi =\frac{\mu_{0}mH}{k_{B}T}$$

and the dipolar coupling parameter

(6) $$\lambda =\frac{\mu_{0}}{4\pi }\frac{m^{2}}{d^{3}k_{B}T}.$$

For a magnetite core with d=10 nm at T=300 K, λ∼1 and ξ=1 corresponds to H≃14 kA m${}^{-1}$ [21]. One more important energy scale is the core anisotropy energy Kv, where *K* is the anisotropy constant of the core material. For iron oxide cores, the anisotropy constant at room temperature is typically assumed to be of the order of $K∼10^{4}$ J/m${}^{3}$ [19,24]. For cores with d=10 nm, $Kv∼k_{B}T$. It was shown in Reference [21] that in this case, the anisotropy effect on the equilibrium magnetic moment and magnetization relaxation of a random cluster is weak and can be neglected. Therefore, magnetic anisotropy is not taken into account here. Note, however, that already at d=20 nm, the anisotropy energy exceeds the thermal one by an order of magnitude, and cannot be ignored (particularly, anisotropy will weaken the cluster magnetic response to a strong field, and will slow down relaxation processes in the system). This situation is beyond the scope of this paper.

**Figure 1:**
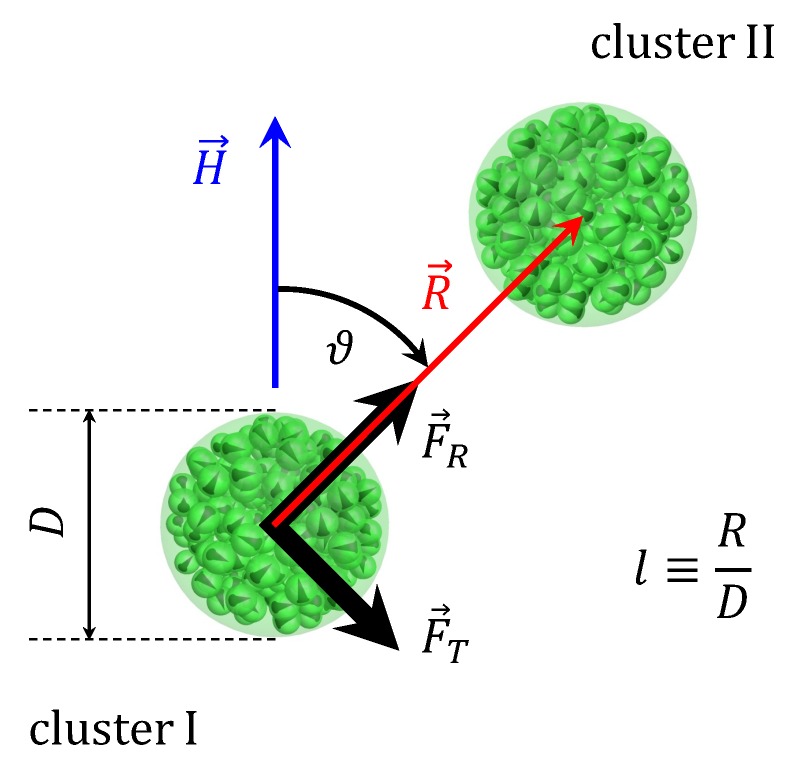
Schematic representation of the problem.

Figure 1 is a schematic representation of the considered problem: an isolated pair of interacting clusters embedded in a non-magnetic medium and subjected to a uniform magnetic field $\vec{H}$. For convenience, let us denote one cluster as “cluster I” and another as “cluster II”. $\vec{R}$ is the vector from the center of the cluster I to the center of the cluster II, and ϑ is the angle between $\vec{H}$ and $\vec{R}$, 0≤ϑ≤π/2. Parameters *N*, φ, λ, and ξ are the same for both clusters. However, the clusters are not identical—each has its own random distribution of cores. The dimensionless quantity $l=|\vec{R}|/D$ is used to characterize intercluster separation. During simulations, translational and rotational movement of clusters is forbidden—only magnetic moments of cores can rotate. To describe their evolution with the allowance of thermal fluctuations and magnetic interactions, the Langevin dynamics simulation method is used. The Langevin equation that describes the magnetodynamics of a single-domain core is the stochastic Landau-Lifshitz-Gilbert equation [46]. For the *i*-th core with magnetic moment ${\vec{m}}_{i}$, it reads

(7) $$\frac{d{\vec{m}}_{i}}{dt}=-\gamma \left[{\vec{m}}_{i}\times {\vec{h}}_{i}^{tot}\right]-\frac{\gamma \alpha }{m}\left[{\vec{m}}_{i}\times \left[{\vec{m}}_{i}\times {\vec{h}}_{i}^{tot}\right]\right],$$

where $\gamma =\gamma_{0}/\left(1+\alpha^{2}\right)$, $\gamma_{0}$ is the gyromagnetic ratio (in meters per ampere per second), α is the dimensionless damping constant, ${\vec{h}}_{i}^{tot}={\vec{h}}_{i}^{det}+{\vec{h}}_{i}^{fl}$, ${\vec{h}}_{i}^{det}=-\left(\partial u_{i}/\partial {\vec{m}}_{i}\right)/\mu_{0}$ is the total deterministic field acting on the core, and ${\vec{h}}_{i}^{fl}$ is the fluctuating thermal field. ${\vec{h}}_{i}^{fl}\left(t\right)$ is a Gaussian stochastic process with the following statistical properties:

(8) $$\langle h_{i,k}^{fl}\left(t\right)\rangle =0,\;\;\langle h_{i,k}^{fl}\left(t_{1}\right)h_{j,l}^{fl}\left(t_{2}\right)\rangle =2D\delta_{ij}\delta_{kl}\delta \left(t_{1}-t_{2}\right),\;\;D=\alpha k_{B}T/\mu_{0}m\gamma \left(1+\alpha^{2}\right),$$

where *k* and *l* are Cartesian indices, $\delta_{ij}$ is the Kronecker delta, δ(t) is the Dirac delta function, and D is the strength of thermal fluctuations. In simulations, the dimensionless form of Equation (7) is used:

(9) $$\begin{array}{c} \frac{d{\vec{e}}_{i}}{d\tau }=-\frac{1}{2\alpha }\left[{\vec{e}}_{i}\times {\vec{\xi }}_{i}^{tot}\right]-\frac{1}{2}\left[{\vec{e}}_{i}\times \left[{\vec{e}}_{i}\times {\vec{\xi }}_{i}^{tot}\right]\right], \end{array}$$

(10) $$\begin{array}{c} {\vec{\xi }}_{i}^{tot}=\frac{\mu_{0}m{\vec{h}}_{i}^{tot}}{k_{B}T}=\xi \hat{\vec{H}}+\lambda \sum_{j,j\neq i}^{2N}\frac{3{\hat{\vec{r}}}_{ij}\left({\vec{e}}_{j}\cdot {\hat{\vec{r}}}_{ij}\right)-{\vec{e}}_{j}}{{\left(r_{ij}/d\right)}^{3}}+{\vec{\xi }}_{i}^{fl}, \end{array}$$

(11) $$\begin{array}{c} \langle \xi_{i,k}^{fl}\left(\tau \right)\rangle =0,\;\;\langle \xi_{i,k}^{fl}\left(\tau_{1}\right)\xi_{j,l}^{fl}\left(\tau_{2}\right)\rangle =\frac{4\alpha^{2}}{1+\alpha^{2}}\delta_{ij}\delta_{kl}\delta \left(\tau_{1}-\tau_{2}\right), \end{array}$$

where ${\vec{e}}_{i}={\vec{m}}_{i}/m$, $\hat{\vec{H}}=\vec{H}/H$, ${\hat{\vec{r}}}_{ij}={\vec{r}}_{ij}/r_{ij}$, $\tau =t/\tau_{D}$, and $\tau_{D}=\mu_{0}m/2\alpha \gamma k_{B}T$ is the characteristic time-scale of the rotary diffusion of the magnetic moment (typically, $\tau_{D}∼10^{-10}$ s [24]). A home-written C++ realization of the standard Heun scheme [46] is used for the numerical integration of Equations (9)–(11). The damping constant in simulations is α=0.1, and the dimensionless integration time-step is ∆τ=0.002. After every time-step, fields ${\vec{\xi }}_{i}^{tot}$ are recalculated using the current orientations of all cores. Dipolar interaction fields between every pair of cores are calculated directly, without any truncations or approximations. The described approach was previously successfully used in Reference [21,25] to study properties of an isolated MCMNP in uniform and constant-gradient magnetic fields.

The main output of the simulations is the equilibrium force acting on Cluster I at given values of *N*, φ, λ, ξ, *l*, and ϑ. Obviously, the force acting on Cluster II has the same magnitude and is of the opposite direction. The force is calculated in the dimensionless form as

(12) $$\frac{\vec{F}D}{k_{B}T}=-3\lambda \frac{D}{d}\langle {\sum_{i=1}^{N}}^{\left(I\right)}{\sum_{j=1}^{N}}^{\left(\mathrm{II}\right)}\frac{{\hat{\vec{r}}}_{ij}\left({\vec{e}}_{i}\cdot {\vec{e}}_{j}\right)+{\vec{e}}_{i}\left({\hat{\vec{r}}}_{ij}\cdot {\vec{e}}_{j}\right)+{\vec{e}}_{j}\left({\hat{\vec{r}}}_{ij}\cdot {\vec{e}}_{i}\right)-5{\hat{\vec{r}}}_{ij}\left({\hat{\vec{r}}}_{ij}\cdot {\vec{e}}_{i}\right)\left({\hat{\vec{r}}}_{ij}\cdot {\vec{e}}_{j}\right)}{{\left(r_{ij}/d\right)}^{4}}\rangle ,$$

where $\sum^{\left(K\right)}$ denotes the sum over cores in the *K*-th cluster. It is conventional to present the force as a sum of two components, $\vec{F}={\vec{F}}_{R}+{\vec{F}}_{T}$ (see Figure 1). The radial component ${\vec{F}}_{R}$ is directed along the center-to-center line, and it is responsible for the repulsion or attraction between clusters. The tangential component ${\vec{F}}_{T}$ is directed perpendicularly to $\vec{R}$, and creates the torque that tends to align the center-to-center line with the field [36]. Angle brackets in Equation (12) denote the averaging over the simulation time. Note that each randomly generated cluster is unique and has its own distinctive spatial distribution of cores. As a result, at given values of *N*, φ, λ, ξ, ϑ, and *l*, different cluster pairs will always give us slightly different magnetic forces. To eliminate this undesirable uncertainty, it is important to simulate many realizations of the same system with different random clusters, and then to additionally average the force values over these realizations. For every set of input parameters, at least ten independent simulations are performed. Error bars on plots below denote 95% confidence intervals for averages over realizations.

## 3. Results and Discussion

### 3.1. Zero-Field Interaction

**Figure 2:**
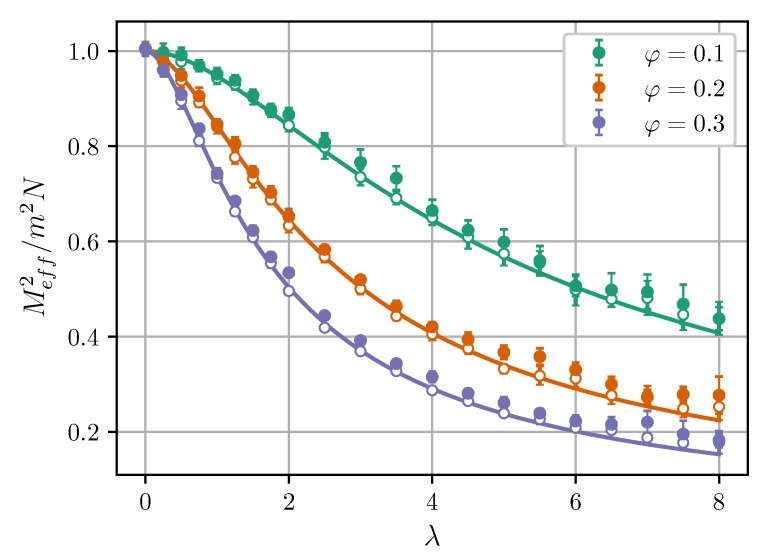
Normalized squared effective magnetic moment of Cluster I vs. the dipolar coupling parameter at ξ=0. Symbols are simulation results for N=100, and curves are from the modified mean-field theory (MMFT) [Equations (15)–(18)]. Empty symbols correspond to l=10, while filled symbols correspond to l=1. Different colors correspond to different volume fractions (see legend).

It is known that even in the absence of an applied magnetic field, MCMNPs have a non-zero effective magnetic moment [20,47]. Our model reproduces this property. Following Reference [20], we define the effective magnetic moment of the cluster as

(13) $$\begin{array}{c} M_{eff}=\sqrt{\langle {\vec{M}}^{2}\rangle }, \end{array}$$

(14) $$\begin{array}{c} \vec{M}=\sum_{i=1}^{N}{\vec{m}}_{i}, \end{array}$$

where $\vec{M}$ is an instantaneous total magnetic moment, and the summation is over all cores in the cluster. Figure 2 shows $M_{eff}^{2}$ (normalized by $m^{2}N$) of Cluster I as a function of λ at different φ, *l*, and ξ=0. Dependencies for Cluster II are equivalent. The effective magnetic moment can also be estimated using a linear response approach [20]:

(15) $$\begin{array}{c} M_{eff}^{2}=\chi_{eff}\frac{3k_{B}TV}{\mu_{0}}=m^{2}N\frac{\chi_{eff}}{\chi_{L}}, \end{array}$$

(16) $$\begin{array}{c} \chi_{L}=\frac{\mu_{0}m^{2}N}{3k_{B}TV}=8\lambda \varphi , \end{array}$$

where $\chi_{eff}$ is the cluster effective magnetic susceptibility, and $\chi_{L}$ is the so-called Langevin susceptibility, which describes a linear magnetic response of an “ideal gas” of non-interacting superparamagnetic cores. It was shown in Reference [21,25] that the magnetic response of an isolated spherical magnetic cluster can be accurately described by the so-called modified mean-field theory (MMFT) [27]. It gives the following expression for the effective susceptibility:

(17) $$\begin{array}{c} \chi_{eff}=\frac{\chi }{1+\chi /3}, \end{array}$$

(18) $$\begin{array}{c} \chi =\chi_{L}\left(1+\chi_{L}/3\right), \end{array}$$

where χ is the magnetic susceptibility of a solid dispersion of interacting superparamagnetic cores.

**Figure 3:**
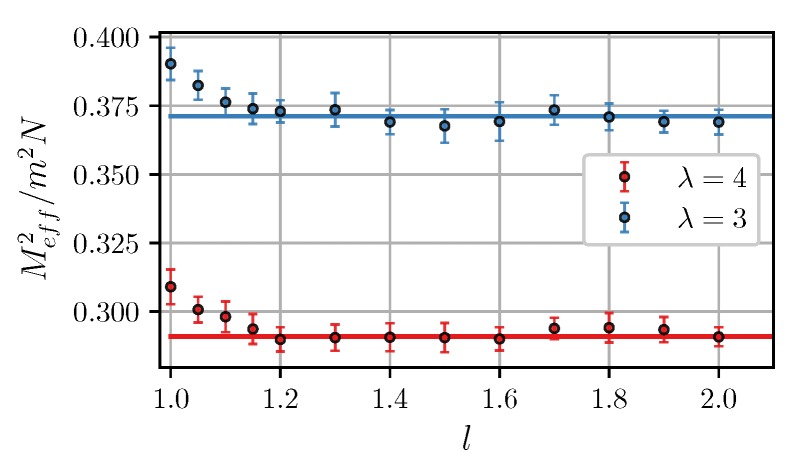
Normalized squared effective magnetic moment of Cluster I vs. the intercluster separation at ξ=0 and φ=0.3. Symbols are simulation results for N=100, and solid lines are from MMFT [Equations (15)–(18)]. Different colors correspond to different λ (see legend).

Figure 2 shows that the simulation data agree with Equations (15)–(18) in a wide range of λ and φ. In the non-interacting limit λ=0, the effective moment equals to the well-known value $mN^{1/2}$ [20,47]. Intracluster dipole-dipole interactions reduce $M_{eff}$. The results also seemingly suggest that effective moments of clusters increase as the intercluster separation decreases, although the effect is rather small. Figure 3 shows the effective magnetic moment as a function of intercluster separation. It is seen that the moment increases at l<1.2.

**Figure 4:**
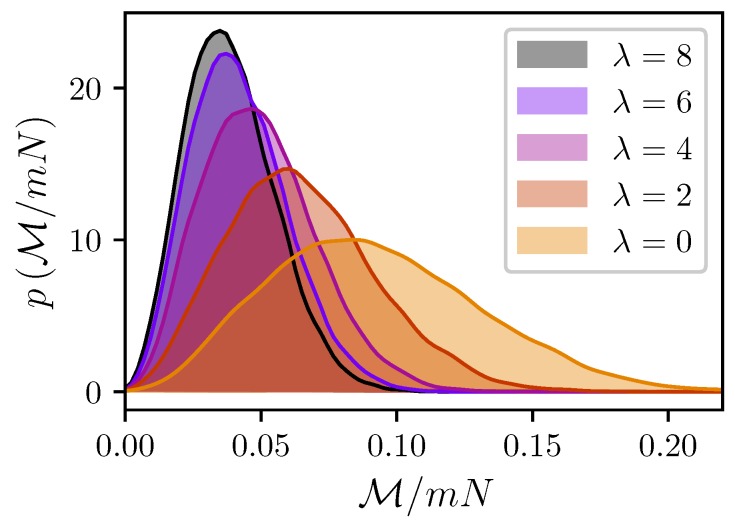
Probability densities for the magnitude of the cluster I magnetic moment. Simulation results for ξ=0, N=100, l=10, and φ=0.3. Different colors correspond to different λ (see legend).

One can reasonably expect that since both clusters have a non-zero effective magnetic moment, the magnetic intercluster force $\vec{F}$ is also non-zero. However, it would be an oversimplification to treat such a zero-field interaction as an interaction between two dipoles with a constant magnitude $M_{eff}$. The cluster magnetic moment $\vec{M}$ is, in fact, a random vector with a strongly fluctuating magnitude, as shown in Figure 4.

Here, the following approach will be used to describe $\vec{F}$ in the absence of a magnetic field. In their seminal paper [48] on magnetic interactions in ferrocolloids, de Gennes and Pincus pointed out that in a zero field, two weakly-coupled cores separated by the distance *r* and subjected to thermal orientational fluctuations will experience an effective attraction, and the corresponding interaction free energy is $w\propto 1/r^{6}$. This interaction is a direct superparamagnetic analog of the van der Waals interaction (more specifically, the Keesom interaction) between two fluctuating polar molecules with a permanent dipole. It seems natural to extend this analogy and to compare the interaction between two clusters filled with fluctuating cores to the van der Waals interaction between two dielectric spheres. The corresponding free energy *W* was given by Hamaker [49]:

(19) $$W\left(l\right)=-\frac{A}{6}\left[\frac{1}{2(l^{2}-1)}+\frac{1}{2l^{2}}+\ln\left(1-\frac{1}{l^{2}}\right)\right],$$

where *A* is the Hamaker constant, which can be found from the Lifshitz theory. If quantum effects are neglected, the Hamaker constant for two spheres in the vacuum is [50]

(20) $$A=\frac{3}{4}k_{B}T\sum_{q=1}^{\infty }q^{-3}{\left(\frac{\chi }{\chi +2}\right)}^{2q},$$

where χ is the static susceptibility of spheres, which, in our case, can once again be taken from MMFT (Equation (18)). The central (attractive) force exerted on Cluster I is

(21) $$\frac{F_{R}D}{k_{B}T}=\frac{\partial }{\partial l}\left(\frac{W}{k_{B}T}\right)=\frac{A}{6k_{B}T}\frac{1}{l^{3}{\left(l^{2}-1\right)}^{2}}.$$

**Figure 5:**
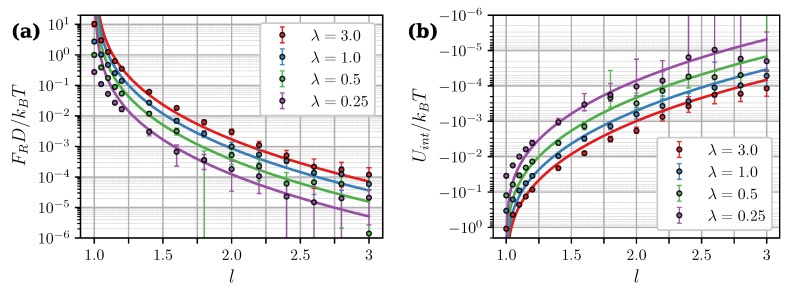
(**a**) Zero-field magnetic force and (**b**) interaction energy of two clusters as a function of the intercluster separation. ξ=0, φ=0.3. Different colors correspond to different λ (see legend). Symbols are simulation results for N=100. Curves are predictions of the Lifshitz theory [Equations (19)–(21)] combined with MMFT [Equation (18)]. An assumption $U_{int}=2W$ was used to obtain theoretical curves in (**b**) [50].

Figure 5a shows simulation results for the zero-field force as a function of intercluster separation at different λ. Theoretical curves from Equation (21) closely follow simulation data at l≳1.2 but significantly overestimate it at smaller separations. In close contact (l=1) the theoretical curves diverge, while in simulations, the force has a well-defined finite value. A similar behavior is observed in Figure 5b, which shows the calculated values of the interaction energy

(22) $$U_{int}=\langle {\sum_{i=1}^{N}}^{\left(I\right)}{\sum_{j=1}^{N}}^{\left(\mathrm{II}\right)}u_{ij}^{dd}\rangle .$$

**Figure 6:**
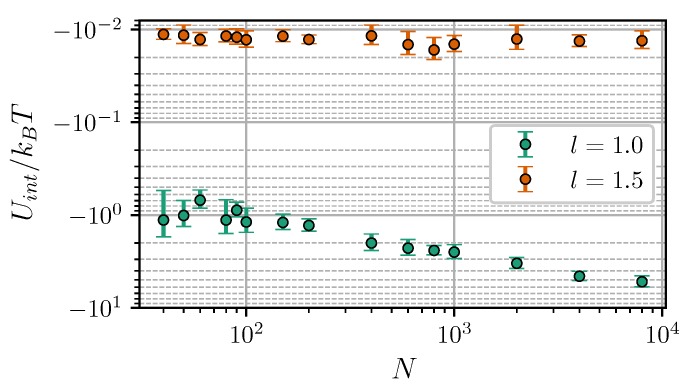
Interaction energy of two clusters vs. the number of cores per cluster. Simulation results for ξ=0, λ=3, and φ=0.3. Different colors correspond to different intercluster separations (see legend).

Note that $U_{int}$, in the general case, does not coincide with the interaction free energy *W*. Let us consider a simple system of two isolated, weakly-coupled fluctuating dipoles of constant magnitude. According to [51], the internal energy of this system $U_{int}=\langle u_{12}^{dd}\rangle$ is connected to the interaction free energy *W* as $U_{int}=2W$. This means that only half of the total energy is available for doing work. The second unavailable half (associated with the entropic contribution to the interaction) is taken up in decreasing the rotational freedom of dipoles and aligning them as they approach each other. To compare the calculated $U_{int}$ with Equations (19) and (20), we used the fact that within the Lifshitz theory, due to its inherent linear response assumption, the relation $U_{int}=2W$ also holds true for a pair of dipole-filled spheres [50]. Presumably, the disagreement between theoretical and numerical results at small separations is a finite-sized effect. This is supported by Figure 6, which shows $U_{int}$ as a function of the core number per cluster *N*. At l=1.5 the energy is almost insusceptible to the cluster size, while in close contact, its absolute value increases almost by an order of magnitude within the investigated range of 40≤N≤8000.

### 3.2. Field-Induced Interaction

In a non-zero field, the interaction between a pair of clusters becomes inherently anisotropic. First, let us analyze the problem within the induced point-dipole approximation, which is often used in the study of magnetorheological fluids and elastomers. It assumes that in a zero-field, there is no interaction between clusters and they both have zero magnetic moments. Under the action of the field, clusters become homogeneously magnetized, and each of them act like a point magnetic dipole $\vec{M}$ (due to the problem symmetry, both clusters have magnetic moments of equal magnitude and direction). The force components can then be written as [36]

(23) $$\begin{array}{c} {\vec{F}}_{R}=\frac{\mu_{0}}{4\pi }\left[\frac{9{\left(\vec{M}\cdot \vec{R}\right)}^{2}}{R^{7}}-\frac{3{\vec{M}}^{2}}{R^{5}}\right]\vec{R}, \end{array}$$

(24) $$\begin{array}{c} {\vec{F}}_{T}=\frac{\mu_{0}}{4\pi }\left[\frac{6{\left(\vec{M}\cdot \vec{R}\right)}^{2}}{R^{7}}\vec{R}-\frac{6(\vec{M}\cdot \vec{R})}{R^{5}}\vec{M}\right]. \end{array}$$

Now, one only needs to find $\vec{M}$. In the weak-coupling limit λ≪1, the mutual magnetization of clusters can be ignored, and the Langevin magnetization law can be used to describe the cluster magnetic response:

(25) $$\begin{array}{c} \vec{M}=mNL\left(\xi \right)\hat{\vec{H}}, \end{array}$$

(26) $$\begin{array}{c} L(\xi )=\coth\xi -1/\xi , \end{array}$$

where L(ξ) is the Langevin function. The force components can then be found analytically:

(27) $$\begin{array}{c} \frac{F_{R}D}{Nk_{B}T}=\frac{3\chi_{L}L^{2}\left(\xi \right)}{8l^{4}}\left(3\cos^{2}\vartheta -1\right), \end{array}$$

(28) $$\begin{array}{c} \frac{F_{T}D}{Nk_{B}T}=\frac{3\chi_{L}L^{2}\left(\xi \right)}{8l^{4}}2\cos\vartheta \sin\vartheta , \end{array}$$

The central force is positive (i.e., attractive) at ϑ=0 (“head-to-tail” configuration), and negative (repulsive) at ϑ=π/2 (“side-by-side” configuration). It changes signs at the critical angle $\vartheta_{0}=\arccos\left(1/\sqrt{3}\right)≃54.7{}^{\circ}$. The tangential force is zero at ϑ=0 and π/2 and has a maximum value at the angle ϑ=π/4, about which it is symmetrical. Further on, Equations (27) and (28) will be referred to as the “Langevin dipoles model” (LDM). For stronger dipolar coupling, the mutual magnetization of clusters can be taken into account by assuming that the total field acting on the cluster is a sum of the applied field and the dipolar field created by the second cluster, i.e.,

(29) $${\vec{H}}_{tot}=\vec{H}+\frac{1}{4\pi }\left[3\frac{\vec{M}\cdot \vec{R}}{R^{5}}\vec{R}-\frac{\vec{M}}{R^{3}}\right].$$

Additionally, the cluster magnetization law at λ>1 has to be changed to take into account the effect of intracluster magnetic interactions. Following Reference [21], we will describe the cluster magnetic response as

(30) $$\vec{M}=mN\psi \left(\frac{\mu_{0}mH_{tot}}{k_{B}T}\right){\hat{\vec{H}}}_{tot}.$$

The function ψ=ψ(x) can be determined from the parametric equation

(31) $$\left\{\begin{array}{c} \psi =L\left(x_{0}+C_{mf}\left(x_{0}\right)L\left(x_{0}\right)\right),\\ x=x_{0}+\chi_{L}\psi , \end{array}\right.$$

where the parameter $x_{0}$ runs from 0 to ∞, and $C_{mf}$ is the mean-field parameter:

(32) $$C_{mf}\left(x_{0}\right)=\chi_{L}\frac{1+a_{2}x_{0}^{2}+a_{4}x_{0}^{4}}{1+b_{2}x_{0}^{2}+b_{4}x_{0}^{4}},$$

$a_{2}$, $a_{4}$, $b_{2}$ and $b_{4}$ are fitting coefficients, which have different values for different combinations of λ and φ (values can be found in Table I of Reference [21]). Thus, a set of Equations (23), (24), (29)–(32) determines the magnetic interaction between two clusters within the induced point-dipole approximation at λ>1. It will be referred to as the “mutual dipoles model” (MDM).

**Figure 7:**
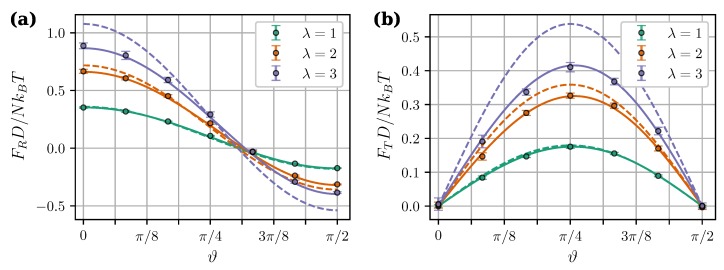
Central (**a**) and tangential (**b**) components of the magnetic intercluster force per core as a function of angle ϑ. φ=0.3, ξ=8, l=1.4. Different colors correspond to different λ (see legend). Symbols are simulation results for N=100, solid curves are from the “mutual dipoles model” (MDM) [Equations (23), (24), (29)–(32)], and dashed curves are from the “Langevin dipoles model” (LDM) [Equations (27) and (28)].

**Figure 8:**
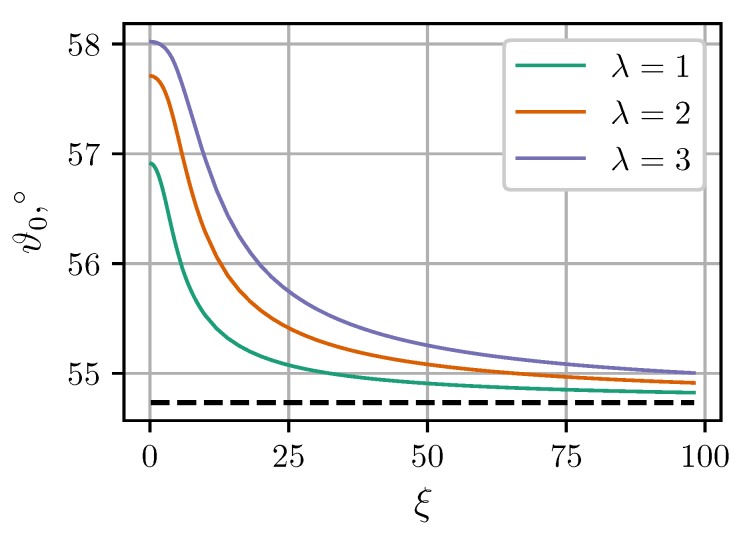
Critical angle $\vartheta_{0}$ at which the central force changes sign as a function of the applied field ξ. Solid curves are MDM predictions for φ=0.3, l=1.4 and different λ (see legend). The dashed horizontal line is the classical LDM value $\vartheta_{0}=\arccos\left(1/\sqrt{3}\right)≃54.7{}^{\circ}$.

Figure 7 shows calculated angular dependencies of the force components at intercluster separation l=1.4 and applied field ξ=8. As expected, MDM predictions at λ>1 are noticeably better than the predictions of LDM. Figure 7 also illustrates two important effects of the mutual magnetization which were previously pointed out and discussed in Reference [36]. Firstly, the angular dependency of the tangential force is no longer symmetric about ϑ=π/4. Secondly, the critical angle $\vartheta_{0}$, at which the central force changes from attractive to repulsive, becomes larger than 54.7°. The latter property is investigated in more detail in Figure 8. It shows the critical angles $\vartheta_{0}$ derived from MDM as a function of the field ξ at different values of λ. The classical value 54.7° plays the role of a strong field asymptote. The angular range of intercluster attraction increases with increasing λ.

**Figure 9:**
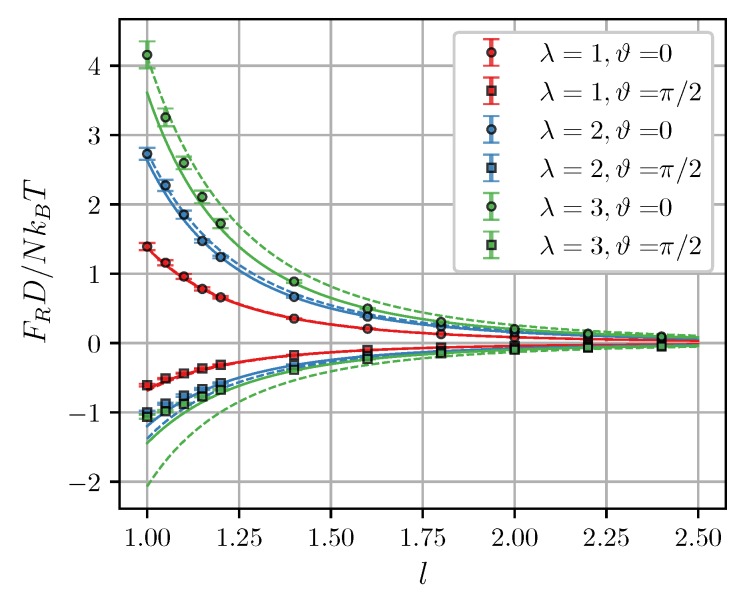
Magnetic central force per core as a function of the intercluster separation at ξ=8 and φ=0.3. Different colors correspond to different λ (see legend). Symbols are simulation results for N=100, circles correspond to the “head-to-tail” configuration (ϑ=0), and squares correspond to the “side-by-side” configuration (ϑ=π/2). Solid curves are corresponding MDM predictions [Equations (23), (29)–(32)], dashed curves are from LDM [Equation (27)].

**Figure 10:**
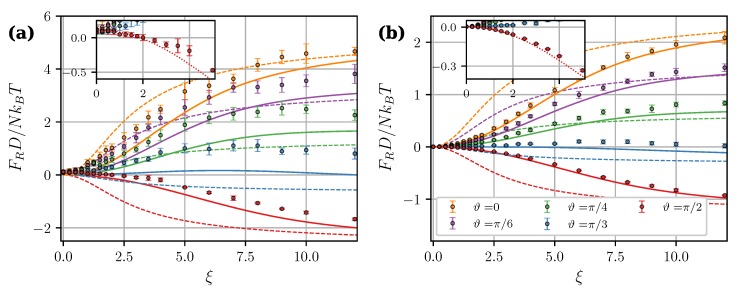
Magnetic central force per core as a function of the applied field ξ at λ=3 and φ=0.3. l=1 (**a**) and 1.2 (**b**). Different colors correspond to different angles ϑ (see legend in (**b**)). Symbols are simulation results for N=100, solid curves are from MDM [Equations (23), (29)–(32)], dashed curves are from LDM [Equation (27)]. Insets show an enlarged view of the weak-field part of simulated dependencies. Dotted lines in insets are MDM predictions for ϑ=π/2 shifted upwards along the *y*-axis to coincide with the simulation data at ξ=0.

Let us now test the MDM applicability limits. In Figure 9, the central force is plotted against separation *l* for the “head-to-tail” and “side-by-side” configurations at ξ=8. In all cases, MDM underestimates the force at l≲1.2. Figure 10 shows the central force as a function of the applied field magnitude ξ at different angles ϑ and separations *l*. Insets in Figure 10 give an enlarged view of the weak-field part of simulated dependencies. The inset in Figure 10a (close contact, l=1) shows that, at all angles, the force tends to a small positive value at ξ=0. Obviously, this limiting value is the van der Waals-like attractive force discussed in the previous section. The most notable case is the dependency for ϑ=π/2: in the “side-by-side” configuration, the force changes from repulsive to attractive at ξ≃2. Below this field, the total magnetic force between two clusters is *always attractive*. It contradicts both MDM and LDM, which always assume zero force in a zero field, and the transition from attraction to repulsion in a non-zero field. In Figure 10b (l=1.2), this effect is much less pronounced, since the zero-field force at l=1.2 drops by more than an order of magnitude, compared to l=1 (see Figure 5a). Dotted curves in insets of Figure 10 are MDM predictions shifted upwards along the *y*-axis to coincide with the simulation data at ξ=0. However, the discrepancy between these curves and simulation points still increases with the field, meaning that the total magnetic force at arbitrary ξ cannot be represented as a simple sum of the zero-field force and the point-dipole force given by Equation (23). A possible reason for this is that, in close contact, the magnetization of clusters is, in fact, non-uniform, and their field-induced properties simply cannot be properly accounted for within any point-dipole approximation [35,36].

## 4. Conclusions

In this work, the Langevin dynamics simulation method was used to calculate the magnetic force between a pair of multicore magnetic nanoparticles (magnetic nanoclusters) embedded in a non-magnetic medium and subjected to a uniform magnetic field. It was found that if clusters are not in close contact (l≳1.2) and if the applied field is strong enough, the force can be described within the induced point-dipole approximation (MDM). However, this approach assumes that there is no zero-field magnetic interaction between clusters. On the contrary, simulation results clearly indicate the presence of an attractive, isotropic, purely magnetic force, even in a zero field. This force is a direct superparamagnetic analog of the van der Waals attraction between two dielectric spheres in a vacuum. For clusters with the number of cores $N∼10^{2}$, this force is much smaller than the force induced by a strong applied field. However, zero-field interaction energy can be comparable with the thermal energy (at λ>1). We thus speculate that this interaction can give rise to magnetically driven self-assembly and phase transformations in MCMNP suspensions. Future work will focus on numerical simulations of large nanocluster ensembles aimed at studying the equilibrium microstructure of an MCMNP suspension and comparing it with the most renown types of magnetic dispersions.

## Abbreviations

The following abbreviations are used in this manuscript:

MCMNP	Multicore Magnetic Nanoparticle
SCMNP	Single-core Magnetic Nanoparticle
MMFT	Modified Mean Field Theory
LDM	Langevin Dipoles Model
MDM	Mutual Dipoles Model
